# Use of transcutaneous electrical nerve stimulation as an adjunctive to epidural analgesia in the management of acute thoracotomy pain

**DOI:** 10.4103/0019-5049.63648

**Published:** 2010

**Authors:** Alka Chandra, Jayant N Banavaliker, Pradeep K Das, Sheel Hasti

**Affiliations:** Department of Anaesthesiology, Rajan Babu Institute of Pulmonary Medicine and TB, New Delhi, India

**Keywords:** Postoperative pain, thoracic epidural analgesia, thoracotomy, transcutaneous electrical nerve stimulation

## Abstract

The present randomized study was conducted in our institute of pulmonary medicine and tuberculosis over a period of 1 year. This study aimed to evaluate the effectiveness of transcutaneous electrical nerve stimulation (TENS) as an adjunctive to thoracic epidural analgesia for the treatment of postoperative pain in patients who underwent posterolateral thoracotomy for decortication of lung. Sixty patients in the age group 15–40 years scheduled to undergo elective posterolateral thoracotomy were divided into two groups of 30 each. Patients were alternatively assigned to one of the groups. In group I, only thoracic epidural analgesia with local anaesthetics was given at regular intervals; however, an identical apparatus which did not deliver an electric current was applied to the control (i.e. group I) patients. While in group II, TENS was started immediately in the recovery period in addition to the epidural analgesia. A 0–10 visual analog scale (VAS) was used to assess pain at regular intervals. The haemodynamics were also studied at regular intervals of 2 h for the first 10 h after the surgery. When the VAS score was more than three, intramuscular analgesia with diclofenac sodium was given. The VAS score and the systolic blood pressure were comparable in the immediate postoperative period (*P* = NS) but the VAS score was significantly less in group II at 2, 4, 6, 8 h (*P* < 0.01, *P* < 0.05, *P* < 0.05, *P* < 0.05, respectively), and at 10 h the *P* value was not significant. Similarly, the systolic blood pressure was significantly less in group II at 2, 4, 6 h after surgery, that is *P* < 0.02, *P* < 0.01, *P* < 0.01, respectively, but at 8 and 10 h the pressures were comparable in both the groups. Adding TENS to epidural analgesia led to a significant reduction in pain with no sequelae. The haemodynamics were significantly stable in group II compared to group I. TENS is a valuable strategy to alleviate postoperative pain following thoracic surgery with no side effects and with a good haemodynamic stability; however, the effects are short lasting.

## INTRODUCTION

Uncontrolled postoperative pain may produce a range of detrimental acute and chronic effects. Attenuation of perioperative pathophysiology that occurs during surgery through reduction of nociceptive input into the central nervous system (CNS) and optimization of perioperative analgesia may decrease complication and facilitate the patients' recovery during the immediate postoperative period and after discharge from the hospital.[1] Epidural analgesia with local anaesthetics and opiod is one of the recommended techniques for the control of postoperative pain. It provides superior postoperative analgesia, limits regression of sensory block and possibly decreases the dose of local anaesthetic administered, although the incidence of side effects may or may not be diminished. In a study by Cooper, when bupivacaine and fentanyl were combined, the sensory block increased but pruritus did not decrease.[2] In order to avoid the side effects of opiods and to limit the dose of local anaesthetics, we decided to add transcutaneous electrical nerve stimulation (TENS) for intensifying the pain relief. Drugs such as clonidine, ketamine, tramadol, fentanyl, midazolam, neostigmine, etc., have been all tried as adjuvants to local anaesthetic agents with varying success rates.[3] This study was designed to evaluate the role of nonpharmacological adjuvant like TENS with epidural analgesia for posterolateral thoracotomies. The objectives of this study were to compare the degree of pain relief, the requirement of additional analgesics and the associated haemodynamic changes by adding TENS. In a study where TENS was used in laminectomy along with morphine, it was found that it offered no advantage over a placebo in the management of acute postoperative pain in the patients.[4] Benedetti *et al*. emphasized that the absence of complications and side effects of TENS compared with conventional opiod and nonopiod analgesics makes electrical stimulation a safe and reliable therapeutic procedure. The TENS treatment was not effective in the posterolateral thoracotomy group, but it was useful as an adjunct to other medications in the muscle-sparing thoracotomy, costotomy and sternotomy groups. TENS is not of major benefit compared with the usual opiod and nonopiod analgesics when pain intensity is high, but it can be used as an adjunct to other medications when the pain is moderate and can be the only pain therapy when the pain is mild.[5] Narcotics used epidurally are associated with undesirable side effects such as respiratory depression, sedation, nausea, vomiting and pruritus. Therefore, adjunctive methods that may limit the narcotic side effects are of considerable interest. TENS has been used to control postoperative pain after various procedures, for example, cardiac operations, cholecystectomy, cesarean section and thoracotomy. The effect of TENS was evaluated in patients of thoracotomy and it was found that TENS group had lower pain scores, shorter recovery stays and better tolerance to chest physical therapy with no respiratory complications.[6]

## METHODS

After obtaining institutional ethical committee approval, 60 patients in the age group 15–40 years of either sex undergoing elective thoracotomies for decortication were included in this study. The patients were randomly divided into two groups of 30 each. One group received only epidural analgesia with local anaesthetics and the other received TENS with epidural analgesia in the postoperative period. Patients with coagulopathy, neurological diseases, spine deformities, diabetes mellitus, pacemaker implantation were excluded from the study.

All the patients were subjected to epidural catheterization before anaesthesia. The catheter was placed in the T7–8 space through 18G Tuohy's needle taking all aseptic and antiseptic precautions. A test dose with 2 ml of 2% lignocaine was given to all the patients. A standard general anaesthetic technique was adopted. Epidural analgesia was given with 10 ml of 0.25% bupivacaine at hourly interval in all the patients intraoperatively. The last dose of analgesia was given 15 min before the closure in all the patients. The patients in both the groups were given epidural analgesia with 10 ml of 0.125% bupivacaine at 2-hourly interval. The time at which the patient was shifted to the recovery room was noted. In the postoperative period, TENS was started immediately in the group II patients by a portable stimulator. The electric current was delivered by two self-adhesive surface electrodes, placed on either side of the incision, approximately 3–4 cm from the suture line. During the 45-min treatment period, the frequency was kept constant at 80 Hz. The patients were shown the visual analog scale (VAS) in the preoperative period. They were asked to quantify their pain using a 10-cm VAS immediately after shifting to the recovery and then at 2, 4, 6, 8 and 10 h after the surgery. An identical apparatus which did not deliver an electric current was applied to the control (i.e., group I) patients. The patients who had a VAS score more than 3 were supplemented with intramuscular diclofenac sodium. The haemodynamics, that is, systolic blood pressure was recorded regularly as planned, and plotted against time.

Side effects, if any, were also observed and noted. The data were analyzed using chi-square test and the *P* values were calculated. *P* < 0.05 was considered significant.

## RESULTS

In this study 60 patients belonging to either sex were divided into two groups of 30 each: group I epidural analgesia and group II epidural analgesia + TENS.

Analysis and comparison are discussed subsequently. As shown in Table 1, patients in both the groups were young and comparable (*P* = NS). The sex ratio in the two groups is shown in Table 2 which is also comparable.

**Table 1:** Mean age in two groups

Age (years)	Group I	Group II
15–20	5	13
21–25	6	6
26–30	7	2
31–35	5	5
36–40	7	4
Mean ± SD	28.7 ± 7.47	25.06 ± 7.32

SD, standard deviation

**Table 2:** Sex ratio in the two groups

Groups	Male	Female	Total
I	15	15	30
II	16	14	30

Time intervals are as follows:

A: Immediate postoperative; B: 2 h after surgery; C: 4 h after surgery; D: 6 h after surgery; E: 8 h after surgery; F: 10 h after surgery.

**Figure 1:**
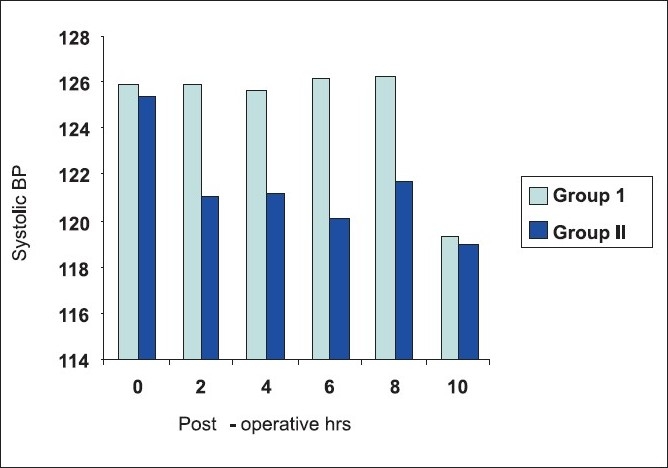
Mean systolic BP plotted against time.

As shown in Table 3, mean basal values of systolic blood pressure in both groups were comparable in the immediate postoperative period. In group II the systolic blood pressure decreased significantly at 2 h (*P* < 0.02), 4 h (*P* < 0.01), 6 h (*P* < 0.01) after the surgery compared to the group where TENS was not applied. However, the blood pressure was comparable at 8 and 10 h in both the groups (*P* = NS) [Figure 1].

**Table 3:** Systolic blood pressure at predefined interval

Time interval	Group I	Group II	*P* value
A	125.80 ± 10.65	125.33 ± 10.05	NS
B	125.80 ± 10.65	121.06 ± 7.42	< 0.02**
C	125.56 ± 10.47	121.20 ± 7.62	< 0.01***
D	126.06 ± 10.56	121.13 ± 7.89	< 0.01***
E	126.20 ± 10.76	121.66 ± 7.41	NS
F	119.33 ± 5.86	118.93 ± 5.57	NS

* *P*< 0.05

** *P*< 0.02

*** *P*< 0.01

It is the degree of significance of the differences in the systolic blood pressure between the two groups where *P*<0.05 is considered significant, NS: Not significant

**Figure 2:**
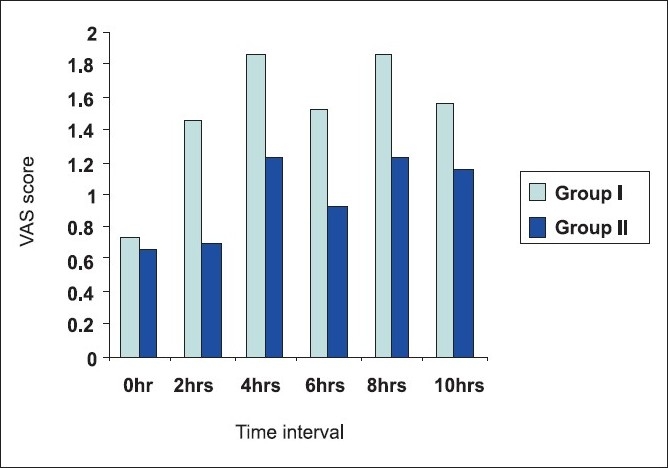
Mean VAS score plotted against time

As shown in Table 4, the values of VAS score were comparable in the immediate postoperative period. However, in group II the pain decreased significantly where TENS was added compared to group I where TENS was not supplemented in the immediate postoperative period. The pain was significantly less at 2, 4, 6 and 8 h after the surgery in group II compared to group I in which only epidural analgesia was given. The pain scores were comparable in both groups at 10 h after surgery [Figure 2].

**Table 4:** VAS score at predefined intervals

Time interval	Group I	Group II	*P* value
A	0.73 ± 0.73	0.66 ± 0.60	NS
B	1.47 ± 0.86	0.70 ± 0.53	< 0.01***
C	1.87 ± 0.82	1.23 ± 0.85	< 0.05*
D	1.53 ± 0.97	0.93 ± 0.78	< 0.05*
E	1.86 ± 0.81	1.23 ± 0.85	< 0.05*
F	1.56 ± 1.10	1.16 ± 1.11	NS

NS: Not significant

* *P*<0.05

** *P*<0.02

*** *P*<0.01

## DISCUSSION

In spite of the great interest in understanding the pain mechanism and pain management, a number of patients suffer from pain even today. So, it is clear that the solution to postoperative pain is not just developing a single technique or a drug to relieve it but to implement simple protocols that suit in different settings with strategies to exploit the available expertise. Anaesthesiologists with their expertise in understanding the pathophysiology of pain, technical expertise and commitment, can organize and manage pain services to the entire hospital.

In the institute where the study was conducted, we get majority of tuberculous patients for thoracotomy. These patients are usually malnourished with restrictive lung patterns in the pulmonary function test and have deranged liver function tests. A thoracotomy is a painful incision as it involves multiple muscle layers, rib resections and continuous motion as the patient breathes. Pain relief is particularly important not only to keep the patient comfortable but also to minimize the pulmonary complications. It enables the patients to ambulate and to breathe normally (without splinting) and to cough deeply.[7] Although it is a routine to give thoracic epidural analgesia with local anaesthetics, the pain relief is not adequate with the local anaesthetics and the patients complain of pain. Increasing the dose of local anaesthetics through thoracic epidural catheter leads to hypotension. Since the hospital lacks the facility of intensive care unit, giving narcotics to the patients who are highly compromised is usually avoided to avoid the side effects of narcotics like nausea, vomiting, respiratory depression, etc. We decided to add TENS instead of narcotics as TENS has also shown to activate the opiod receptors and it is without any side effects and suits our setup.

Though pharmacotherapy forms an integral part of management of acute pain, one has to look at various other methodologies to relieve postoperative pain. The nonpharmacological methods can be used as adjuvants to the main method of pain relief. A holistic approach can cut down costs and reduce the complications associated with the opiod and nonopiod drug usage and dosage. A lot of interest is being shown on complimentary and alternative medicine for chronic pain management, which could be applied for acute pain. Herbal medicine, hypnosis, homeopathy, therapeutic touch, meditation, TENS, acupuncture, heat application are a few to name. It is accepted that use of TENS either at acupoint or dermatome corresponding to surgical incision decreases the postoperative pain, opiod requirement and the related side effects. The effect of frequency of electrical stimulation was studied in 100 women undergoing gynecological surgeries. The application of TENS showed better pain relief and 55% decrease in morphine requirement, as well as reduced nausea and vomiting. The mechanism by which TENS produces analgesia is unclear and may be related to the modulation of nociceptive impulses in the spinal cord, release of endogenous enkephalins or a combination of these and other mechanisms.[8] Although the analgesic efficacy of these techniques are controversial, TENS and acupuncture may provide postoperative analgesia, decrease postoperative opiod requirements, reduce opiod-related side effects and attenuate sympathoadrenal system activation.[5,9]

TENS has been hypothesized to improve pain in multiple ways. Theories include effects on sensory nerves, interference with sensory discriminative pathways, stimulation of release of natural chemicals that affect the way pain is perceived and transmitted, for example, enkephalins and endorphins or through increased blood flow in treated areas such as the skin or heart.[10] Recent data suggest that pain relief from low- and high-frequency TENS is mediated by the release of mu- or delta-opiods in the CNS and reductions in substance P. It is also sometimes suggested that TENS affects the cardiovascular system, increasing the heart rate and reducing the blood pressure.[11]

In a controlled trial of TENS for postoperative pain relief following inguinal herniorraphy, pain was assessed over the first three postoperative days by VAS, expiratory peak flow rates and analgesics requirements. There was no difference between the two groups for pain scores and it was concluded that TENS does not reduce postoperative pain. However, it had considerable patient appeal and many patients believed that it was effective.[12] Sodipo *et al*.[13] proved that for patients given TENS along with narcotics immediately after surgery, there is a marked significant decrease in the amount of narcotics administered. There was favorable nursing, physician and patient acceptance to these devices. Treatment of pain after thoracotomy is important not only to ensure patient comfort but also to minimize pulmonary complications. Erdogan *et al*. have proved in their study that TENS is beneficial for pain releif following thoracotomy and has no side effects and hence recommended the routine use of TENS following thoracic surgery.[14]

Thoracic epidural analgesia is the gold standard for post-thoracotomy analgesia. Local anaesthetics given epidurally can supplement general anaesthesia during surgery, but postoperatively their addition may not significantly improve analgesia above the level achieved from epidural opiods only. Any concurrent hypotension and motor blockade from the local anaesthetics can limit the patients' ability to ambulate. Local anaesthetics can cause postoperative hypotension which is as high as approximately 7%, the average may be closer to 0.7–3%.[15] Hence, to limit the dose of local anaesthetics and to avoid the side effects of opiods like nausea, vomiting, pruritus, etc., we decided to supplement epidural analgesia with TENS. TENS has been used as an adjunct to narcotic analgesics for improving the outcome of thoracic surgery and it not only has shown benefits in the treatment of acute thoracotomy pain but also when used together with narcotics, reduces the duration of recovery room stay and increases chest physical tolerance with positive effects on pulmonary functions. Hence, current evidence shows that TENS associated with postoperative medications is safe and effective in alleviating postoperative pain and improving patient recovery, thus enhancing the choice of available medical care and bettering the outcome of thoracic surgery.[16] In our study, epidural analgesia was given 10 min before the closure of incision in both the groups with 10 ml of 0.25% bupivacaine and the pain was assessed immediately after shifting the patients to the recovery. In groups I and II, the doses of epidural with 10 ml of 0.125% bupivacaine were given at interval of 2 h and pain was assessed 15 min after the dose using VAS score in both the groups. However, in group II, TENS was added immediately in the recovery room. The haemodynamics, that is, blood pressure and pulse rate were recorded at 2-hourly interval when the pain was assessed. The systolic blood pressure was comparable in both the groups immediately after surgery but the pressures reduced significantly in group II at 2 h (*P* < 0.02), 4 h (*P* < 0.01) and 6 h (*P* < 0.01). However, the blood pressures were comparable in both the groups at 8 and 10 h (*P* = NS). The patients having VAS score of >3 were supplemented with an intramuscular dose of diclofenac sodium. The VAS score of 0 was considered as no pain, 1 as mild, 2 as moderate pain and 3 or more as severe. The VAS score was similar in both the groups in the immediate postoperative period (*P* = NS). At 2, 4, 6 and 8 h, the score was significantly lower in the group II patients (*P* < 0.01, *P* < 0.05, *P* < 0.05, *P* < 0.05). Again, at 10 h, the pain score was comparable in both the groups. The incidence of side effects was negligible in both the groups. The acceptance to chest physiotherapy in the patients who received TENS was more than the patients who did not receive it. The satisfaction level of the patients who received TENS was also more compared to those who did not receive it.

## CONCLUSION

Addition of TENS to epidural analgesia by local anaesthetics intensifies the pain relief without causing any squeals in addition to stabilizing the haemodynamics. TENS can be used as an adjunctive to epidural analgesia for acute postoperative pain in patients of posterolateral thoracotomies.

## References

[1] Holte K, Kehlet H (2002). Effect of postoperative epidural analgesia on surgical outcome. Minerva Anestesiol.

[2] Cooper DW, Ryall DM, Hardy FE, Lindsay SL Eldabe SS (1996). Patient-controlled extradural analgesia with bupivacaine, fentanyl or a mixture of both, after caesarian section. Br j Anaesth.

[3] de Beer DA, Thomas ML (2003). Caudal additives in children-solutions or problems?. Br J Anaesth.

[4] McCallum MI, Glynn CJ, Moore RA, Lammer P, Phillips AM (1988). Transcutaneous electrical nerve stimulation in the management of acute postoperative pain. Br J Anaesth.

[5] Benedetti F, Amanzio M, Casadio C, Cavallo A, Cianci R, Giobbe R (1997). Control of postoperative pain by transcutaneous electrical nerve stimulation after thoracic operations. Ann Thorac surg.

[6] Warfield CA, Stein JM, Frank HA (1985). The effect of transcutaneous electrical nerve stimulation on pain after thoracotomy. Ann Thorac Surg.

[7] Gerner P (2008). Post-thoracotomy pain management problems. Anesthesiol Clin.

[8] Hamza MA, White PF, Ahmed HE, Ghoname EA (1999). Effect of the frequency of transcutaneous electrical nerve stimulation on the postoperative opioid analgesic requirement and recovery profile. Anesthesiology.

[9] Kotani N, Hashimoto H, Sato Y, Sessler DI, Yoshioka H, Kitayama M (2001). Preoperative intradermal acupuncture reduces postoperative pain, nausea and vomiting, analgesic requirement and sympathoadrenal responses. Anesthesiology.

[10] Bushnell MC, Marchand S, Tremblay N, Duncan GH (1991). Electrical stimulation of peripheral and central pathways for the relief of musculoskeletal pain. Can J Physiol Pharmacol.

[11] Campbell TS, Ditto B (2002). Exaggeration of blood pressure-related hypoalgesia and reduction of blood pressure with low frequency transcutaneous electric nerve stimulation. Psychophysiology.

[12] Gilbert JM, Gledhill T, Law N, George C (1986). Controlled trial of transcutaneous electrical nerve stimulation (TENS) for postoperative pain relief following inguinal herniorrhaphy. Br J Surg.

[13] Sodipo Jo, Adedeji SA, Olumide O (1980). Postoperative pain relief by transcutaneous electrical nerve stimulation (TENS). Am J Chin Med.

[14] Erdogan M, Erdogan A, Erbil N, Karakaya HK, Demircan A (2005). Prospective, Randomized, Placebo-controlled Study of the Effect of TENS on postthoracotomy pain and pulmonary function. World J Surg.

[15] Wheatley RG, Schug SA, Watson D (2001). Safety and efficacy of postoperative epidural analgesia. Br J Anaesth.

[16] Freynet A, Falcoz PE (2010). Is transcutaneous electrical nerve stimulation effective in relieving postoperative pain after thoracotomy?. Interact Cardiovasc Thorac Surg.

